# Resolution of Disseminated Molluscum Contagiosum and Kaposi Lesions With Highly Active Antiretroviral Therapy (HAART) in an HIV Patient

**DOI:** 10.7759/cureus.79686

**Published:** 2025-02-26

**Authors:** Mouna Guechchati, Meryem Soughi, Hamza Idrissi Janati, Fatima Zahra Mernissi, Samira Rabhi

**Affiliations:** 1 Dermatology Department, University Hospital Hassan II, Faculty of Medicine, Pharmacy and Dental Medicine, Sidi Mohamed Ben Abdellah University, Fez, MAR; 2 Infectious Diseases Department, University Hospital Hassan II, Faculty of Medicine, Pharmacy and Dental Medicine, Sidi Mohamed Ben Abdellah University, Fez, MAR

**Keywords:** haart, hiv, kaposi sarcoma, molluscum contagiosum, resolution

## Abstract

Dermatologic disorders are common in people living with HIV (PLHIV), with Kaposi’s sarcoma (KS) and molluscum contagiosum (MC) being strongly associated with immune status. We report the case of a 48-year-old male individual newly diagnosed with HIV, presenting with necrotizing cytomegalovirus retinopathy and tuberculosis. He developed multiple erythematous-violaceous macules on the trunk, arms, and face, confirmed as KS on biopsy, along with numerous umbilicated papules consistent with MC. His initial CD4 count was 31/mm³. The patient was started on highly active antiretroviral therapy (HAART) without additional interventions. Within six months, complete resolution of both KS and MC lesions was observed, with a CD4 count increase to 218/mm³. Two years post-diagnosis, the patient remains in remission with stable immune function. This case highlights the efficacy of HAART alone in treating both viral and tumoral dermatologic manifestations in HIV patients. Early initiation of HAART plays a pivotal role in disease resolution and overall prognosis.

## Introduction

More than 90% of people living with HIV (human immunodeficiency virus) (PLHIV) will develop at least one dermatologic disorder during the course of their infection, ranging from common inflammatory conditions to rare opportunistic infections. Dermatologic manifestations can serve as early indicators of HIV infection, and some conditions are so closely tied to immune status that their presence can define specific stages of HIV or signify progression to AIDS (acquired immune deficiency syndrome). Opportunistic infections, such as Kaposi’s sarcoma (KS) and molluscum contagiosum (MC) [1], are strongly associated with advanced immunosuppression, particularly in patients with low CD4 counts [2]. The advent of highly active antiretroviral therapy (HAART) in the mid-1990s has significantly altered the epidemiology and clinical spectrum of HIV-related skin disorders, leading to improved immune recovery and spontaneous resolution of certain lesions [1]. However, the response of specific dermatologic conditions to HAART alone varies, highlighting the importance of understanding their natural progression and treatment outcomes.

We report the case of a patient with a newly diagnosed HIV infection, which revealed KS and multiple MC lesions. The evolution of these lesions was favorable with antiretroviral therapy alone.

## Case presentation

A 48-year-old male individual with a medical history of type 2 diabetes was diagnosed with HIV in September 2022, presenting with complications of necrotizing CMV retinopathy and tuberculosis, which led to the discovery of HIV.

He was referred to our department due to the recent emergence of multiple skin lesions involving the face, neck, and body.

**Figure 1:**
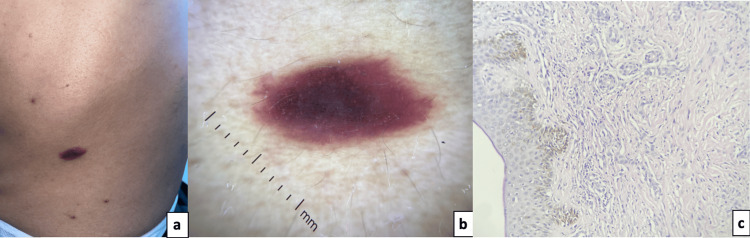
Oval-shaped erythematoviolet plaque on right flank (a), homogeneous purple-reddish pattern (b), and slit-like vascular channels infiltrating dermis with infiltrative proliferation of spindled endothelial cells (c).

On examination, two types of lesions were identified. Erythematous-violaceous macules were observed on the trunk, arms, and face (Figure 1a), with dermoscopy findings suggestive of KS (Figure 1b).

**Figure 2:**
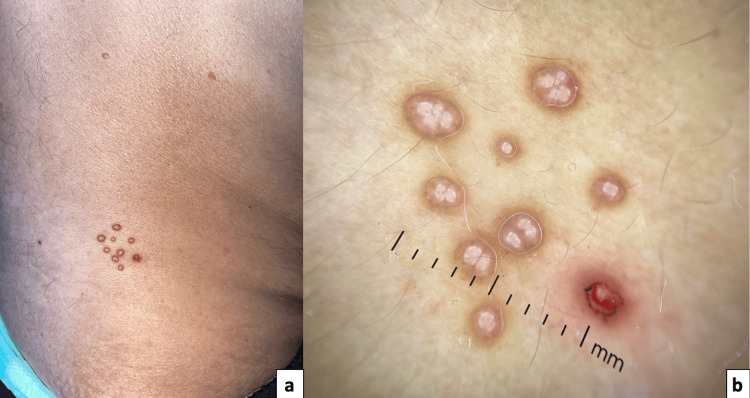
Skin-colored papules with central umbilication, some clustered and others diffusely scattered across the body (a), and dermoscopy revealing a clover-like pattern with amorphous white-yellowish structures and crown-like vascularization (b).

A skin biopsy confirmed the diagnosis (Figure 1c), with no systemic involvement identified on staging. Additionally, multiple skin-colored papules with central umbilication, some clustered and others diffusely scattered across the body, were noted (Figure 2a). Dermoscopy revealed a clover-like pattern with amorphous white-yellowish structures and crown-like vascularization (Figure 2b), confirming MC.

At the initiation of HAART, the patient’s CD4 count was 31/mm³. The patient was prescribed emollient creams along with HAART, consisting of two nucleoside reverse transcriptase inhibitors (lamivudine and tenofovir) and an integrase inhibitor (dolutegravir), with close monitoring.

**Figure 3:**
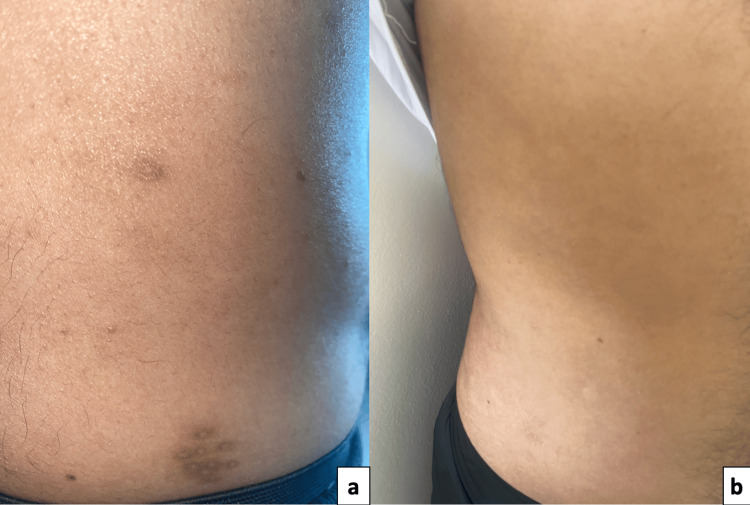
Regression after three months of treatment with persistence of pigmented macula (a), and complete disappearance after six months (b).

After three months, a marked improvement in both conditions was observed (Figure 3a). By six months, the patient’s CD4 count had increased to 218/mm³, with complete resolution of KS and MC achieved (Figure 3b), without the need for additional interventions.

Two years post-diagnosis, the patient remains in remission, with stable immune function, adherence to HAART, and maintaining body weight.

## Discussion

Skin diseases are common features of HIV patients, varying by geographic region and the availability of HAART [3]. The introduction of HAART in the mid-1990s has significantly reduced the prevalence of AIDS-defining illnesses, including KS while influencing the prevalence of other HIV-related skin disorders such as MC [1].

KS remains the most common HIV-related malignancy globally, classifying it as a Stage C condition according to the CDC classification; although its incidence has significantly decreased in developed countries since the advent of HAART [4]. The mechanism behind this lies in the ability of HAART to suppress viral replication and improve immune function, reducing the immunodeficiency that underpins KS development [5]. In this case, the patient’s KS lesions responded completely to HAART within six months, underscoring the importance of immune restoration in halting KS progression, even in the absence of systemic involvement.

MC, on the other hand, caused by a poxvirus, is typically self-limiting in immunocompetent individuals, with lesions resolving within six to 12 months. However, in immunosuppressed patients, including those living with HIV, MC often presents with numerous, atypical lesions resistant to conventional therapies [6]. Improvement in immune function through HAART has been reported as the mainstay for MC resolution in such cases [7]. Hurni et al. documented the complete disappearance of MC lesions in HIV patients using a combination of saquinavir, zidovudine, and lamivudine, while other reports highlight similar outcomes with different ART regimens [7]. In our case, the use of dolutegravir, lamivudine, and tenofovir led to the resolution of MC lesions within six months, supporting the pivotal role of HAART in managing this viral infection.

This case is particularly notable for the rapid and concurrent resolution of both KS and MC, two distinct HIV-related conditions, without recurrence over a two-year follow-up period. Such findings align with previous reports suggesting that immune reconstitution via HAART alone can effectively treat both viral and tumoral skin pathologies in HIV patients [8].

## Conclusions

This case underscores the efficacy of HAART as a standalone treatment for both Kaposi's sarcoma and molluscum contagiosum in HIV patients. The rapid and sustained resolution of both conditions highlights the critical importance of immune restoration in addressing diverse HIV-related dermatologic manifestations.
